# Evidence of Big Five and Aggressive Personalities in Gait Biomechanics

**DOI:** 10.1007/s10919-016-0240-1

**Published:** 2016-09-06

**Authors:** Liam Satchell, Paul Morris, Chris Mills, Liam O’Reilly, Paul Marshman, Lucy Akehurst

**Affiliations:** 1 Department of Psychology, University of Portsmouth, King Henry Building, King Henry 1 Street, Portsmouth, PO1 2DY UK; 2 Department of Sport and Exercise Science, University of Portsmouth, Cambridge Road, Portsmouth, PO1 2ER UK

**Keywords:** Gait biomechanics, Trait aggression, Big Five personality

## Abstract

Behavioral observation techniques which relate action to personality have long been neglected (Furr and Funder in Handbook of research methods in personality psychology, The Guilford Press, New York, 2007) and, when employed, often use human judges to code behavior. In the current study we used an alternative to human coding (biomechanical research techniques) to investigate how personality traits are manifest in gait. We used motion capture technology to record 29 participants walking on a treadmill at their natural speed. We analyzed their thorax and pelvis movements, as well as speed of gait. Participants completed personality questionnaires, including a Big Five measure and a trait aggression questionnaire. We found that gait related to several of our personality measures. The magnitude of upper body movement, lower body movement, and walking speed, were related to Big Five personality traits and aggression. Here, we present evidence that some gait measures can relate to Big Five and aggressive personalities. We know of no other examples of research where gait has been shown to correlate with self-reported measures of personality and suggest that more research should be conducted between largely automatic movement and personality.

## Introduction

…the sense I have of my own importance is carried in the way I swagger. Indeed, some of the most pervasive features of my attitude to the world and to others is encoded in the way I project myself in public space; whether I am macho, or timid, or eager to please, or calm and unflappable. (Taylor 1995, p. 171)

The philosopher Taylor (1995) suggests that there is a relationship between his ‘attitude’ and the way he walks. However, Taylor’s claim has not been subject to thorough empirical investigation. If Taylor is correct, the automatic action of walking could contain information about personality traits. We know of only one other study that has explored this interesting question and in their investigation, Thoresen et al. (2012) did not find a relationship between gait and Big Five personality traits. It is possible that Thoresen et al. did not find a relationship between personality and gait due to their choice of gait analysis. Thoresen et al. adapted Troje’s (2002) technique of reducing whole body motion down to a smaller number of parameters for analysis. This method of analysis does not include the information available from examining relative movements of the body, such as relative upper body to lower body motion. In our experiment, we utilized gait analysis techniques typical of the gait literature (such as, Goujon-Pillet et al. 2008) that include this information which could demonstrate the relationship between gait and personality.

Behavioral observation is an established research technique used (albeit normally for actions more deliberate or more automatic than walking) in individual differences psychology for judging personality from action. It has been claimed that behavioral observation has been neglected as a research technique in trait psychology experiments for some time (Furr and Funder 2007). Behavioral observation techniques require a coder to rate participants’ actions and behaviors, such as facial movement (Ekman et al. 2002) or ‘social engagement’ (Funder et al. 2000) for analysis. However, using human coders to recognize the ‘relevant’ (Funder 1999) cues to personality has room for error, as some important detail of movement may not be salient or of interest to a human coder. Here, we capture the movements of individuals with standard gait biomechanics measures and attempt to demonstrate a relationship between movement and personality.

Our intention was to deliver proof of concept research that demonstrates the relationship between gait and personality. We focused on the relationship between the Big Five (using the measure created by John et al. 2008) and gait. We also investigated the relationship between gait and dispositional aggression (using the measure created by Buss and Perry 1992). It would be beneficial if there were cues to an approaching stranger’s inclination to aggression in their gait. Further, Troscianko et al. (2004) encourage the investigation of any potential relationship between an individual’s biological motion and their intention to engage in aggression. Their research finds that ‘distinctive gait’ is important for predicting impending criminal acts but Troscianko et al. (2004) do not provide analyses to demonstrate what this distinctive gait may be.

The current research was exploratory with no firm hypotheses. Instead, we explored various aspects of gait and their relationships with aggression and big five personality traits. It is reasonable to expect some relationships between sex-typical personality and sex-typical motion, such as expecting the known differences in trait aggression (with men typically being more aggressive, Eagly and Steffen 1986; Wilson and Daly 1997) to relate to the sex typical upper body or ‘thorax’ movement (with men typically having more upper body rotation within the horizontal plane and women having more pelvis rotation within the horizontal plane, Bruening et al. 2015). Thus, it is possible, for example, that those in our sample with greater thorax movement (which is seen to be male-typical) will report male-typical high trait aggression. However, it is difficult to predict how movement variation *within* the sexes would relate to traits, and therefore we form no specific hypotheses.

## Method

### Participants

Twenty-nine participants were recruited (male = 16, *M*_*Age*_ = 21.14 years, *SD*_*Age*_ = 2.28 years) for this exploratory research. Participants were given a £5 shopping voucher for their participation.

### Materials

Participants completed the Buss–Perry Aggression Questionnaire (Buss and Perry 1992, analysed using revisions suggested by Bryant and Smith 2001) and the Big Five Inventory (John et al. 2008). The Buss–Perry Aggression Questionnaire quantifies trait aggression into four subscales; tendencies to physical aggression and Verbal aggression and dispositional anger and hostility towards others. The Buss–Perry Aggression Questionnaire is a commonly used psychometric tool to measure trait aggression has good evidence of external validity (Archer and Webb 2006; Diamond 2006; O’Connor et al. 2001). The Big Five Inventory is a measure of the widely used Big Five traits; Conscientiousness, Agreeableness, Neuroticism, Openness to Experience and Extraversion. The Big Five model is the most widely accepted and researched personality model in individual differences research (Funder 2001).

### Procedure

**Fig. 1:**
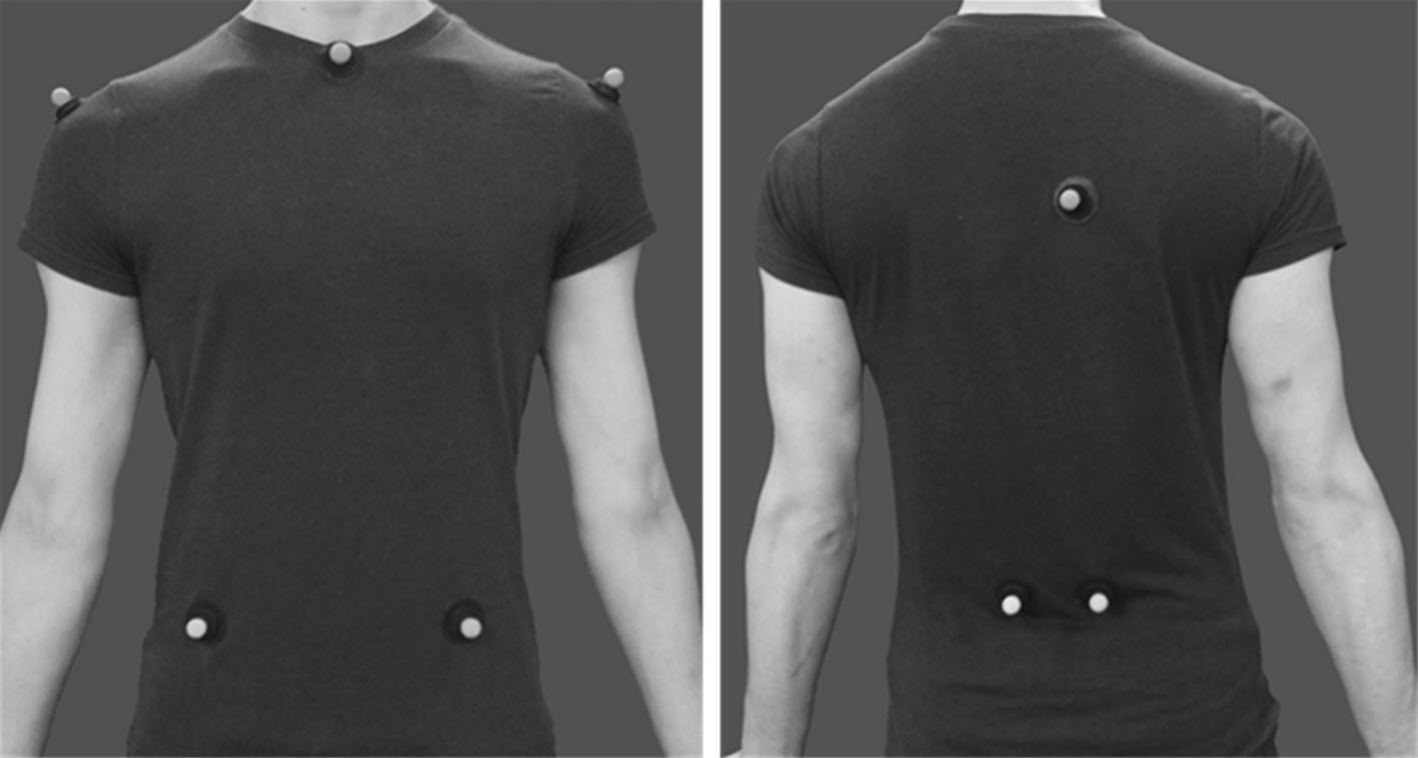
*Left* the anterior view of marker placements, showing retroreflective markers placed on the acromion process of both shoulders (thorax reference point), the suprasternal notch (thorax reference point) and the crests of the anterior iliac spines (pelvis reference point). *Right* the posterior view of marker placements, showing retroreflective markers placed on the posterior crests of the iliac spines (pelvis reference point) and a tracking marker placed right of the spine on the upper back (overall reference point)

Once participants had been informed and given the opportunity to consent on the required ethical and medical information, participants completed a self-directed familiarization trial on the treadmill (Powerjog, Germany). This time allowed participants to become familiar walking on a treadmill and to find a speed of walk that they found familiar. Following the familiarization trial reflective markers (12 mm diameter) were attached to specific anatomical positions on the thorax (used to track the motion of the upper body) and pelvis (used to track the lower body) of the participants (Fig. 1). Additional markers on the legs and feet were used to identify walking gait cycles throughout the trials. Three dimensional movement of the markers only (similar to point light models), were tracked by 16 Oqus 3+ cameras (Qualisys, Sweden), sampling at 200 Hz, positioned in an arc around the treadmill. Cameras were calibrated using a coordinate frame positioned on the treadmill and a hand held wand containing markers of predefined distances [for our motion capture software; Qualisys Track Manager (QTM)].

Before the gait could be measured, the participants stood still in the anatomical position (a standard body pose for biomechanical and physiological measurement) for 10 s, in order to have the data to create a model of the body for later use in gait analyses. Participants were asked to adjust the treadmill speed to that of their normal gait (gait speed in km/h) from their experience in the familiarization trial. When participants felt ready, the motion capture equipment recorded their walking gait for 60 s. Subsequently, participants completed the Buss–Perry Aggression Questionnaire and the Big Five Inventory and were debriefed.

### Analyses

#### Statistical Analyses

As aspects of movement may not be normally distributed we opted to use nonparametric analyses for our results; Mann–Whitney *U* tests for the demonstration of sex differences and Spearman’s rank-order correlations to demonstrate the relationship between psychometrics and gait. Nonparametric tests are better suited to analysing smaller sample sizes, such as those in this exploratory research.

#### Gait Analyses

**Fig. 2:**
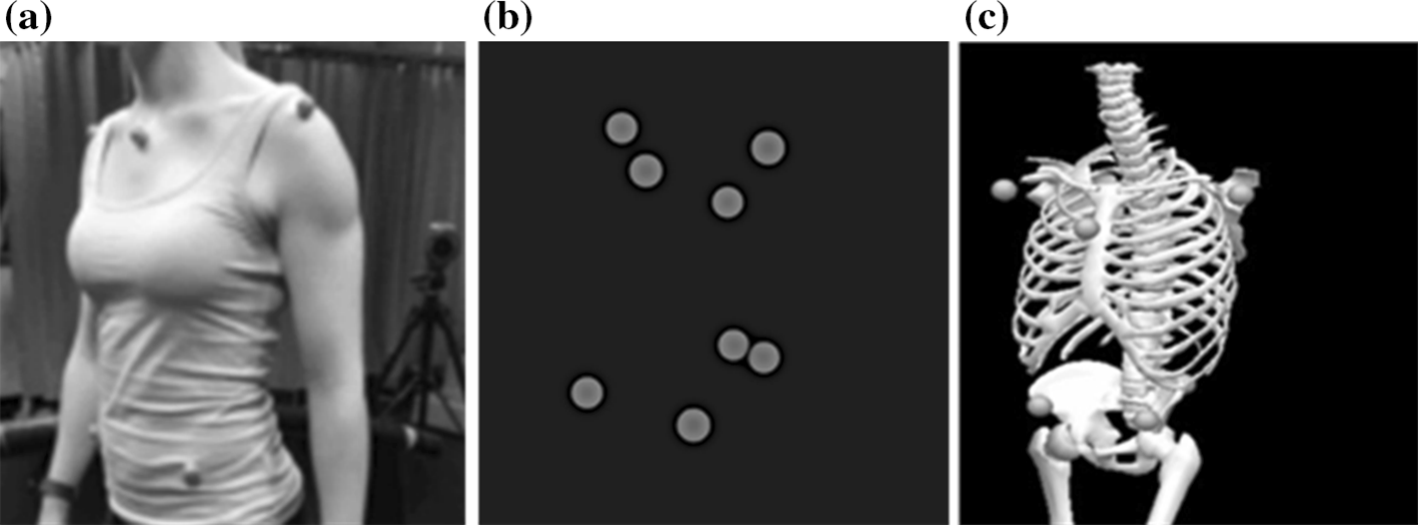
A participant presented as; **a** photograph of in the laboratory on the treadmill, **b** the measured location of the reflective markers in three-dimensional space [presented in Qualisys Track Manager (QTM)] and **c** a three-dimensional model based on the identified markers, ready for analysis [presented in V3D (C-Motion Inc., Germantown, USA)]

Motion capture systems, such as the one we use for analysis, record the movement of the reflective markers placed on the participants (see Fig. 2a), individually, in three-dimensional space. Then, using specialised software (QTM), the three-dimensionally measured reflections from the markers have to be manually identified as the associated anatomical mark of the walking person (see Fig. 2b). This allowed us to create the three-dimensional models of our participants, represented as X, Y and Z dimensional data. Then, the reconstructed marker positional data from both the static and walking trials were imported in Visual 3D (C-Motion Inc., Germantown, USA) to construct a thorax and pelvis model for each participant (see Fig. 2c). We then produced a customized data processing script (computer code) to find the first five uninterrupted gait cycles (as defined by change in the velocity of heel markers, see Zeni et al. 2008) out of the 60 s walk, and used for analysis.

**Fig. 3:**
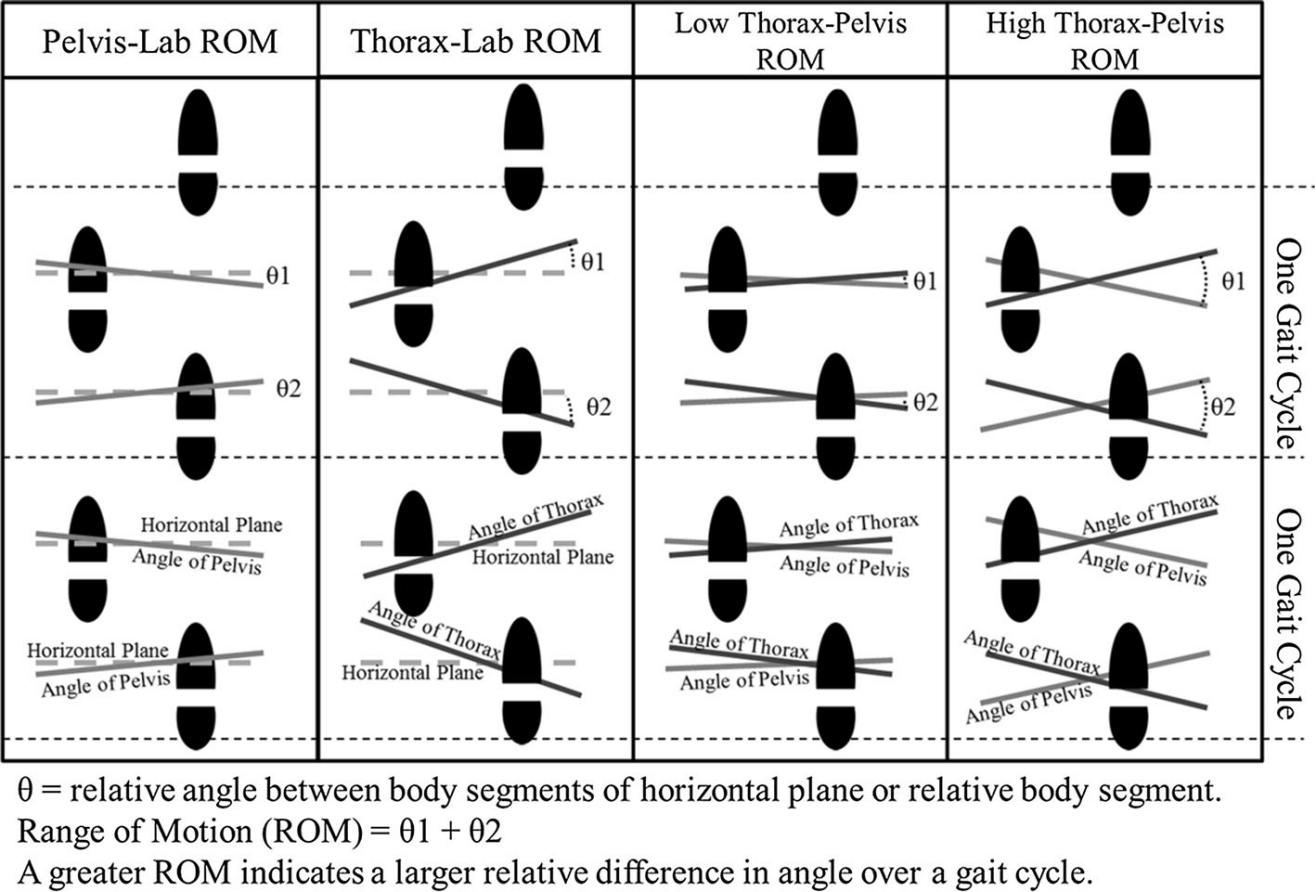
A graphical representation of the range of motion measurements used in this experiment

The customized data processing script was also used to calculate the key gait characteristics within this study. We calculated the range of motion (ROM) of the thorax relative to the fixed horizontal plane, known as ‘the laboratory’ (Thorax-Lab ROM, see Fig. 3), to give a measure of upper body movement. We also calculated the range of motion of the pelvis relative to the fixed horizontal plane (Pelvis-Lab ROM, see Fig. 3) for a measure of lower body movement. Further, we calculated the range of motion of the thorax (upper body) relative to the pelvis (lower body) in the horizontal plane (Thorax–Pelvis ROM, see Fig. 3). From each gait cycle, ranges of motion (ROM) were calculated by finding the difference between the minimum and maximum angle of movement during each gait cycle. The ROM was averaged across the five gait cycles to produce a single value for analysis per participant. These measures are typical of research in gait biomechanics (see similar analyses by Goujon-Pillet et al. 2008).

## Results

### Sample Sex Differences in Personality and Gait

We report some notable sex differences in the biomechanical (such as, Thorax–Pelvis ROM) and psychometric (such as, Neuroticism and Hostility) measures (see Table 1) of our study, but none of these were moderate or large effects (see Ferguson 2009). Due to the evidence of sex differences from our own sample, as well as of the known sex differences in gait style (Bruening et al. 2015; Troje 2002) and physical aggression (Eagly and Steffen 1986; Wilson and Daly 1997) in the existing literature, we chose to analyze the data from females and males separately in addition to analyzing the pooled data.

**Table 1:** The Mann–Whitney *U* differences between sexes for the biomechanical and psychometric measures

Variable	*U*	Cliff’s δ	*p*	Female participants		Male participants	
				Mean	*SD*	Mean	*SD*
Thorax–Pelvis ROM (°)	58.00	.41	.044	7.71	2.94	5.63	2.06
Thorax-Lab ROM (°)	77.00	.25	.236	8.28	2.34	7.35	2.16
Pelvis-Lab ROM (°)	68.00	.34	.114	7.22	1.89	5.95	1.92
Gait speed (km/h)	98.50	.06	.809	3.40	0.73	3.44	0.71
Physical aggression	74.50	.28	.195	24.69	7.62	28.81	9.17
Verbal aggression	60.50	.42	.056	14.69	4.48	18.38	5.57
Anger	85.00	.18	.403	22.15	4.06	21.44	6.67
Hostility	52.00	.50	.022	29.31	8.89	21.31	8.91
Conscientiousness	93.50	.11	.644	31.54	5.63	32.19	4.53
Agreeableness	76.00	.25	.217	35.00	4.65	33.56	3.31
Neuroticism	42.50	.59	.007	27.77	4.11	19.44	8.92
Openness	79.50	.24	.280	34.15	4.34	32.13	5.08
Extraversion	95.50	.04	.714	25.85	4.04	25.31	6.76

Ranges of motion are as follows; Thorax–Pelvis is the range of thorax motion relative to the participants’ pelvic motion, Thorax-Lab is the range of thorax motion relative to the horizontal plane (laboratory) and Pelvis-Lab range is the range of pelvic motion relative to the horizontal plane (laboratory)

### Aggression and Motion Correlations

We tested for Spearman’s rank-order correlations between biomechanical aspects of gait and trait aggression. The results showed that participants’ Thorax–Pelvis ROM reflected the trait physical aggression of individuals, especially with female participants (see Table 2). It should be noted that it was only the relative motion of thorax and pelvis that correlated with aggression; neither thorax movement on its own nor pelvis movement on its own correlated with aggression. Rather, the increased relative movement of the thorax (upper body) and pelvis (lower body) together was reflective of increased physical aggression. There was a moderate correlation between gait speed and aggression for males but no correlation between gait speed and aggression for females or the sample as a whole.

**Table 2:** Spearman’s ranked correlations between biomechanics and psychometrics across participants split by gender and together as an overall sample (with *p* values in brackets)

Trait	Ranges of motion^a^			
	Thorax–Pelvis	Thorax-Lab	Pelvis-Lab	Gait speed
Male participants (*N* = 16)				
Physical aggression	0.47 (.066)	0.39 (.130)	0.26 (.332)	0.33 (.211)
Verbal aggression	0.42 (.106)	**0.72 (.002)**	**0.67 (.004)**	−0.47 (.065)
Anger	0.34 (.201)	0.35 (.182)	0.43 (.095)	0.35 (.180)
Hostility	−0.23 (.383)	0.17 (.532)	0.31 (.250)	−0.37 (.159)
Conscientiousness	−0.31 (.235)	−0.18 (.516)	−0.12 (.665)	0.07 (.801)
Agreeableness	0.05 (.856)	−0.01 (.969)	0.01 (.974)	−0.02 (.952)
Neuroticism	−0.23 (.397)	−0.23 (.387)	−0.09 (.736)	−0.21 (.429)
Openness	−0.23 (.391)	−0.42 (.106)	−0.29 (.274)	0.17 (.526)
Extraversion	0.11 (.692)	**0.58 (.018)**	0.49 (.051)	0.11 (.697)
Female participants (*N* = 13)				
Physical aggression	**0.74 (.004)**	0.03 (.914)	−0.20 (.502)	−0.07 (.821)
Verbal aggression	0.08 (.802)	−0.16 (.614)	−0.13 (.679)	−0.05 (.878)
Anger	0.55 (.051)	0.06 (.837)	−0.12 (.693)	0.17 (.590)
Hostility	−0.02 (.943)	−0.42 (.149)	−0.47 (.102)	−0.22 (.478)
Conscientiousness	−0.21 (.499)	−**0.69 (.009)**	0.14 (.641)	0.55 (.054)
Agreeableness	0.06 (.843)	−0.47 (.102)	**0.80 (.001)**	**0.64 (.017)**
Neuroticism	0.01 (.971)	0.33 (.271)	−0.28 (.360)	−0.44 (.133)
Openness	0.23 (.443)	−0.43 (.145)	0.02 (.957)	−0.03 (.928)
Extraversion	0.26 (.385)	−0.37 (.219)	0.54 (.055)	0.51 (.075)
Overall sample (*N* = 29)				
Physical aggression	**0.43 (.019)**	0.23 (.232)	0.02 (.939)	0.12 (.550)
Verbal aggression	0.08 (.697)	0.20 (.305)	0.18 (.344)	−0.19 (.312)
Anger	0.48 (.008)	0.26 (.170)	0.21 (.285)	0.27 (.163)
Hostility	0.09 (.641)	0.02 (.940)	0.07 (.712)	−0.31 (.099)
Conscientiousness	−0.21 (.285)	−**0.52 (.004)**	0.06 (.765)	0.39 (.036)
Agreeableness	0.06 (.750)	−0.20 (.310)	0.48 (.008)	0.29 (.122)
Neuroticism	0.01 (.944)	0.05 (.786)	0.01 (.964)	−0.27 (.158)
Openness	0.03 (.884)	−0.28 (.141)	−0.07 (.722)	0.03 (.863)
Extraversion	0.13 (.489)	0.18 (.362)	**0.43 (.019)**	0.23 (.233)

Bold type highlights effects that are significant at *p* < .05

^a^Ranges of motion are as follows; Thorax–Pelvis range is the range of Thorax movement relative to the participants’ range of pelvic movement, Thorax-Lab range is the range of Thorax movement relative to the lab and Pelvis-Lab range of motion is the range of pelvic movement relative to the lab

### Big Five Factors and Motion Correlations

There were some elements of gait that were powerfully correlated with the Big Five traits (using Spearman’s ranked correlations, see Table 2). Notably large correlations included the strong positive correlations between agreeableness and Pelvis-Lab ROM (.80) and conscientiousness and Thorax-Lab ROM (−.69) for female participants, and extraversion and Thorax-Lab ROM (.58) for male participants.

## Discussion

Taylor (1995) claimed that the “swagger” (p. 171) of his walk reflected his personality. In our exploratory biomechanical analysis of gait, we provide empirical evidence that suggests that personality related to how a person walks. Aspects of gait, such as (a) relative movement between the upper and lower body (Thorax–Pelvis ROM), (b) upper body movement alone (Thorax-Lab ROM), (c) lower body movement alone (Pelvis-Lab ROM), and (d) gait speed, related to differing aspects of our participants’ personality. Importantly, our use of biomechanics allowed a measurement approach to analyzing gait that was not reliant on human coders. However, this technology does record more information than a human could process (our equipment captures movement at 200 Hz), suggesting that our results may present the available cues to personality in biomechanical recording and not the available cues to personality in visual perception. This concern was also raised by Thoresen et al. (2012) which is why they chose to analyze their movement based on what was visually salient. In our study, we opted to use the gait analysis more typical of the biomechanics literature as we are interested in the relationship between the actual movement involved in gait and personality (rather than how people make judgements about gait), which may explain the difference between our findings and those of Thoresen et al. We also examined different aspects of movement, such as our Thorax–Pelvis ROM, instead of Thoresen et al.’s compound whole body measures, which may have allowed us further sensitivities to gait mechanics relevant to our measured traits.

The walks of participants who self-reported high physical aggression comprised greater relative movement between the upper and lower body (higher Thorax–Pelvis ROM). Importantly, this relationship was not simply a property of heightened shoulder movement (Thorax-Lab ROM) or pelvis movement (Pelvis-Lab ROM) as these variables alone were not predictive of aggression; it was the relative motion of the upper and lower body that was important. When walking, the body naturally rotates a little; as an individual steps forward with their left foot, the left side of the pelvis will move forward with the leg and the left shoulder will move back and the right shoulder forward to maintain balance. Put simply, an aggressive walk is one where this rotation is exaggerated. These findings support Troscianko et al.’s (2004) suggestion that there are aspects of biological motion that could be useful to detect in crime prevention. If the closed circuit television (CCTV) observers in their experiments could be trained to recognize the aggression-relevant gait demonstrated in this research, their ability to recognize impending crimes (which is already high) could be improved further.

It is relatively easy to account for the relationships between gait and self-reported aggression. However, the relationships found between gait and big five traits are more difficult to explain. The relationships we might have expected, such as a strong positive relationship between extraversion and amount of movement in gait, were not consistently found; moreover, the largest correlations observed were between aspects of gait and conscientiousness and gait, and agreeableness and gait. From a theoretical perspective it would be easy to explain how extraversion might be available to the perceiver in gait, but more challenging to explain how conscientiousness and agreeableness might be available.

It is plausible that gait affects personality through the embodiment of a walking style, for example, adopting a confident style of gait and then self-rating high extraversion (see similar work on ‘Power poses’, Cesario and McDonald 2013). Further research is required to establish whether gait affect personality or personality affects gait. It could also be the case that gait affects how participants complete self-report measures, with feelings of aggression or confidence (neuroticism or extraversion) being ameliorated or diminished by recently having their gait observed.

Our findings of correlations between gait and personality are interesting, as gait is a behavior that holds an unusual place between the automatic, physiological correlates of personality (such as, cerebral blood flow; Johnson et al. 1999) and the behavioral or social engagement correlates of personality (such as, ‘acting playfully’; Funder et al. 2000). To most people walking is a relatively automatic behavior yet it is reflective of individual psychology, such as one’s openness to new experiences. Our findings are important in explaining accurate interpersonal trait judgments and our objective methods could be used with other potentially personality-relevant movements, such as seated pose and shaking hands. The existence of these correlations demonstrates the potential of research into relationships between individual differences in psychology and individual differences in movement.

## Conclusion

In this exploratory paper, we found a large number of strong correlations between various aspects of gait and pen and paper psychometric measures. This suggests that there can be relationships between walking style and personality. We avoid extensive theoretical interpretation of the particular relationships between aspects of gait and personality traits revealed in the study, instead our conclusion is a broad one; personality is manifest in the way we walk.
